# Public health round-up

**DOI:** 10.2471/BLT.25.010125

**Published:** 2025-01-01

**Health care under attack in Lebanon**

Staff working in the burns unit of the Geitaoui Hospital, Beirut, Lebanon, a country where more health workers and patients have been killed proportionally in the ongoing conflict than in any active conflict today across the globe – with nearly half of all attacks on health care causing the death of a health worker or patient.

**Figure:**
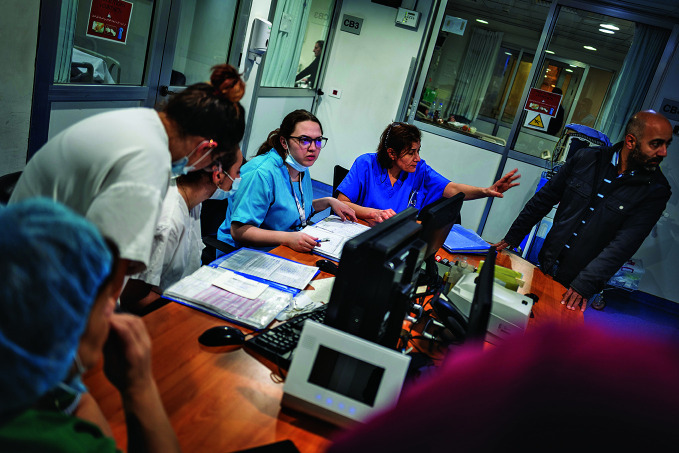
WHO/Hedinn Halldorsson

## WHO supports investigation of undiagnosed disease

Between 24 October and 5 December 2024, Panzi health zone in Kwango Province of the Democratic Republic of the Congo recorded 406 cases of an undiagnosed disease with symptoms of fever, headache, cough, runny nose and body ache.

According to the World Health Organization’s (WHO) disease outbreak news report of 8 December, all the severe cases were people reported to be severely malnourished. Among those people, 31 were reported to have died. The majority of cases reported are among children, particularly those under five years of age.

WHO sent experts to the Democratic Republic of the Congo to support efforts to determine the cause of the disease. They joined the national rapid response team comprised of epidemiologists, clinicians, laboratory technicians, infection prevention and control and risk communication experts. A local WHO team had already been supporting the health authorities in Kwango since the end of November to reinforce disease surveillance and identify cases.

https://bit.ly/4g6exFb

https://bit.ly/4glU5Qh

## Malaria cases rise

In 2023, there were an estimated 263 million new malaria cases in 83 countries worldwide, up from 252 million in 2022 and 226 million in 2015. This according to the *World malaria report 2024* which was released on 11 December. Malaria incidence also rose, growing from 58 to 60.4 cases per 1000 population at risk in the period 2015–2023.

The African Region remains hardest hit by malaria, accounting for an estimated 94% of global cases and 95% of malaria-related deaths in 2023. Just over half of these deaths occurred in four countries: Nigeria (30.9%), Democratic Republic of the Congo (11.3%), Niger (5.9%) and United Republic of Tanzania (4.3%). The global tally of malaria deaths reached 597 000 in 2023 compared to 578 000 in 2015.

Progress towards key targets of the WHO global malaria strategy, which calls for reductions in malaria incidence and death rates of at least 75% by 2025 and 90% by 2030 compared to 2015 baseline levels, remains substantially off track.

https://bit.ly/3DaXR0G

## Mpox spreads

The number of mpox cases continues to rise and spread, especially those due to monkeypox virus clade Ib infection. These were among the key conclusions of the International Health Regulations (2005) Emergency Committee on mpox in 2024, convened on 22 November.

Highlighting the need for stronger national response efforts as well as a sustained cohesive response across countries and partners, the Committee advised that the event continues to meet the criteria for a public health emergency of international concern (PHEIC). The WHO Director-General concurred with the advice and issued revised temporary recommendations.

In his 28 November virtual press conference, WHO Director-General Tedros Adhanom Ghebreyesus noted that as of that date, 20 countries in Africa had reported more than 14 000 confirmed cases, more than 75% of which were in the Democratic Republic of the Congo.

In related news, WHO granted Emergency Use Listing for the LCI16m8 mpox vaccine, the second mpox vaccine to be listed for emergency use.

https://bit.ly/3B92vfc

https://bit.ly/4g8bigG

https://bit.ly/3ZocjKl

## Geographical expansion of Oropouche

Cases of Oropouche virus infection expanded across the Region of the Americas, with three new countries – Ecuador, Guyana, and Panama – and the Cayman Islands reporting confirmed cases between 23 August and 25 November 2024. Imported cases were also identified in Canada, the United States of America and parts of the European Region.

According to WHO’s disease outbreak news report of 5 December, a total of 11 634 confirmed cases, including two deaths, had been reported across 10 countries and one territory. The outbreak primarily affected regions where the disease is endemic, such as Bolivia's La Paz department and Brazil's Amazon region, but new transmissions emerged in previously unaffected areas.

Oropouche virus disease is mainly spread to humans through the bite of the *Culicoides paraensis* midge, commonly found near forests and water, or certain *Culex quinquefasciatus* mosquitoes. The symptoms resemble dengue, and include sudden fever, severe headaches, joint pain, chills and nausea, lasting up to seven days.

https://bit.ly/3Bw41YJ

## Casualties in the Lebanon conflict

More health workers and patients have been killed proportionally in Lebanon than in any active conflict today across the globe – with nearly half of all attacks on health care causing the death of a health worker or a patient.

According to a 22 November WHO media release, presenting the latest reporting by WHO’s Surveillance System for Attacks on Health Care (SSA), since 7 October 2023, 47% of attacks on health care – 65 out of 137 – have proven fatal to at least one health worker or patient in Lebanon, as of 21 November 2024.

According to the SSA, 226 health workers and patients were killed in Lebanon and 199 injured between 7 October 2023 and 18 November 2024. In the same period, the SSA registered a combined total of 1264 attacks on health care in the occupied Palestinian territory (oPt), and Israel – 1196 in the oPt and 68 in Israel.

“These figures reveal yet again an extremely worrying pattern. Depriving civilians of access to lifesaving care and targeting health providers is a breach of international humanitarian law,” said WHO Representative in Lebanon Dr Abdinasir Abubakar.

https://bit.ly/3ZHAGUF

## Preventing health-care-associated infections

Progress in addressing critical gaps to prevent health-care-associated infections is lagging behind targets set. This was among the key findings of a new global report on infection prevention and control (IPC) launched by WHO on 29 November at a Group of 7 side-event hosted by Italy.

Providing a baseline assessment for policy-makers, IPC professionals, health workers and stakeholders to guide action, the report finds that although 71% of countries now have an active IPC programme, just 6% met all of the WHO IPC minimum requirements in 2023–2024. This is well behind the target of more than 90% by 2030 set in the WHO Global action plan and monitoring framework on IPC.

The report also highlights the fact that patients in low- and middle-income countries have up to 20 times higher risk of acquiring infections during health-care than in high-income countries.

https://bit.ly/49qYbEw

## Towards sustainable health-care facilities

WHO released guidance on what is needed to develop safe, climate-resilient and environmentally sustainable health-care facilities to provide quality care and withstand environmental crises.

Aimed at health-care facility managers, health workers and national authorities, the guidance provides an overview and definitions of safe, climate-resilient and environmentally sustainable health-care facilities, and lists concrete guidance about actions that can be taken to achieve them. The guide also points to key materials, resources and tools that provide more detailed guidance and actions.

https://bit.ly/3OKLDyo

**Cover photo**

A mounted part-time health worker delivers vaccination and other health services in the Karatal Japyryk area of the Naryn region, Kyrgyzstan.

**Figure:**
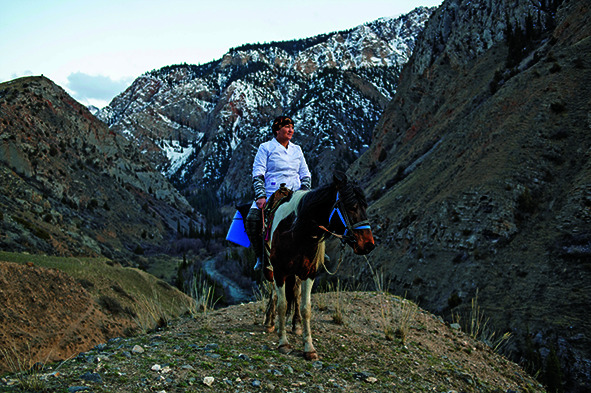
(WHO/Danil Usmanov)

## The genital herpes burden

An estimated 846 million people aged between 15 and 49 are living with genital herpes infections – more than 1 in 5 of this age-group globally, according to a study released on 11 December.

Genital herpes infection can lead to painful genital sores and blisters that can recur throughout life, causing significant discomfort and often requiring multiple health-care visits. 

According to the study, at least 42 million people acquire a new genital herpes infection each year, while more than 200 million people aged 15 to 49 suffered at least one symptomatic episode in 2020.

The authors of the study, which was published in the journal *Sexually Transmitted Infections*, say that new treatments and vaccines are needed to reduce the impact of the herpes virus and control its spread.

https://bit.ly/3ZLMLrL

## Funding WHO

WHO’s inaugural Investment Round reached a high point at the G20 Leaders' Summit in Rio de Janeiro, Brazil on 19 November. With the pledges received from Australia, Indonesia and Spain at the Leaders’ Summit, and the United Kingdom of Great Britain and Northern Ireland shortly afterwards, WHO has now received pledges of 1.7 billion United States dollars.

https://bit.ly/3D7ignk

**Looking ahead**

23–24 January 2025. International Conference on Global Health. London, United Kingdom. https://bit.ly/48PzwZW

28 January–2 February 2025. Prince Mahidol Award Conference 2025. Bangkok, Thailand. https://bit.ly/3AGb5BS

4 February 2025. World Cancer Day: United by Unique. Events worldwide. https://bit.ly/49K7XlD

